# Long-term retention on treatment with lumiracoxib 100 mg once or twice daily compared with celecoxib 200 mg once daily: A randomised controlled trial in patients with osteoarthritis

**DOI:** 10.1186/1471-2474-9-32

**Published:** 2008-03-07

**Authors:** Roy Fleischmann, Hyman Tannenbaum, Neha P Patel, Marianne Notter, Peter Sallstig, Jean-Yves Reginster

**Affiliations:** 1 The University of Texas Southwestern Medical Center at Dallas, MCRC, Dallas, Texas, USA; 2 Rheumatic Disease Center of Montréal, Montréal, Canada; 3 Novartis Pharmaceuticals Corporation, East Hanover, NJ, USA; 4 Novartis Pharma AG, Basel, Switzerland; 5 CHU Policlinique L. Brull, Liege, Belgium

## Abstract

**Background:**

The efficacy, safety and tolerability of lumiracoxib, a novel selective cyclooxygenase-2 (COX-2) inhibitor, has been demonstrated in previous studies of patients with osteoarthritis (OA). As it is important to establish the long-term safety and efficacy of treatments for a chronic disease such as OA, the present study compared the effects of lumiracoxib at doses of 100 mg once daily (o.d.) and 100 mg twice daily (b.i.d.) with those of celecoxib 200 mg o.d. on retention on treatment over 1 year.

**Methods:**

In this 52-week, multicentre, randomised, double-blind, parallel-group study, male and female patients (aged at least 40 years) with symptomatic primary OA of the hip, knee, hand or spine were randomised (1:2:1) to lumiracoxib 100 mg o.d. (n = 755), lumiracoxib 100 mg b.i.d. (n = 1,519) or celecoxib 200 mg o.d. (n = 758). The primary objective of the study was to demonstrate non-inferiority of lumiracoxib at either dose compared with celecoxib 200 mg o.d. with respect to the 1-year retention on treatment rate. Secondary outcome variables included OA pain in the target joint, patient's and physician's global assessments of disease activity, Short Arthritis assessment Scale (SAS) total score, rescue medication use, and safety and tolerability.

**Results:**

Retention rates at 1 year were similar for the lumiracoxib 100 mg o.d., lumiracoxib 100 mg b.i.d. and celecoxib 200 mg o.d. groups (46.9% vs 47.5% vs 45.3%, respectively). It was demonstrated that retention on treatment with lumiracoxib at either dose was non-inferior to celecoxib 200 mg o.d. Similarly, Kaplan-Meier curves for the probability of premature discontinuation from the study for any reason were similar across the treatment groups. All three treatments generally yielded comparable results for the secondary efficacy variables and all treatments were well tolerated.

**Conclusion:**

Long-term treatment with lumiracoxib 100 mg o.d., the recommended dose for OA, was as effective and well tolerated as celecoxib 200 mg o.d. in patients with OA.

**Trial registration:**

clinicaltrials.gov NCT00145301

## Background

Osteoarthritis (OA) is the most common arthritis in adults, with an estimated worldwide prevalence of 9.6% for men and 18.0% for women aged at least 60 years [1]. The prevalence of OA is particularly high in Europe and the USA compared with other parts of the world. As a consequence of an increasingly aging population together with an elevated risk for OA with advancing age, OA will become an even greater burden in the coming years.

OA is a major cause of impaired mobility that has a serious detrimental impact on a patient's quality of life and their ability to perform normal daily activities [2,3]. Indeed, it is associated with a substantial non-fatal burden of disease, estimated to account for 2.8% of total years of living with disability [4]. OA is characterised by joint pain, tenderness, stiffness, crepitus and local inflammation [1] and most commonly affects the joints of the hip, knee, hand, foot and spine.

The pharmacological options for treating OA pain include simple analgesics (e.g. acetaminophen/paracetamol), traditional nonselective nonsteroidal anti-inflammatory drugs (NSAIDs) and selective cyclooxygenase-2 (COX-2) inhibitors [3,5]. Although the efficacy of traditional NSAIDs for relieving OA pain is well established [6,7], they can be associated with serious gastrointestinal (GI) complications [8,9]. NSAID-related gastric damage is caused by inhibition of cyclooxygenase-1 (COX-1) and prostaglandins, which help to maintain GI mucosal integrity. Hence, selective COX-2 inhibitors have been developed to try to minimise these potentially serious GI effects while providing analgesia comparable with traditional NSAIDs [10-14].

Lumiracoxib (Prexige^®^) is a novel selective COX-2 inhibitor developed for the treatment of OA [14-18] and acute pain, such as dental pain following surgery [19], sprains and strains [20], primary dysmenorrhoea [21] and acute flares of gout [22].

As a chronic disease, OA often requires continued management for extended periods and it is important to establish the safety and efficacy of treatments in the long term [3,5]. Moreover, it is difficult to keep many patients on their OA medications and the majority of patients discontinue treatment after 1 year [23]. Hence, the objective of this 52-week study was to investigate the long-term effects of lumiracoxib 100 mg o.d. (the recommended dose in OA) and lumiracoxib 100 mg twice daily (b.i.d.) compared with celecoxib 200 mg o.d. in patients with OA of the knee, hand, hip or spine. In order to provide an indication of overall effectiveness and safety, the primary outcome variable used in this study was retention on treatment. Retention on treatment reflects the interrelated issues of efficacy, safety and tolerability and represents a relevant measure for assessment in clinical trials.

## Methods

### Study design

This was a 52-week, multicentre, randomised, double-blind, double-dummy, active-controlled, parallel-group study conducted in 65 centres in Europe (Belgium, Germany, France, Italy and Switzerland) and 149 centres in North America (Canada and the USA). The study received Ethics Committee approval, was performed in accordance with the ICH Harmonized Guidelines for Good Clinical Practice (GCP) and the Declaration of Helsinki, and all patients provided written, informed consent before the start of the study.

Following a 3–7-day screening period, during which the patient's current NSAID therapy was washed out, patients were randomised (1:2:1) to lumiracoxib 100 mg o.d., lumiracoxib 100 mg b.i.d. (twice the recommended dose in OA) or celecoxib 200 mg o.d. for 52 weeks. Since there was limited experience with the lumiracoxib 100 mg b.i.d. dose, twice as many patients were recruited into this treatment arm.

After giving written informed consent, patients were assigned a unique patient identification number. Randomisation was performed using an interactive voice response system which collected patient identifying information by telephone and assigned randomisation numbers linking the patient to a treatment group and specifying a unique medication number for the study drug to be dispensed to the patient. A validated and automated system assigned patient numbers to randomisation numbers in a block formation in order to ensure treatment groups were balanced within centres. A separate medication randomisation list was produced using a validated system that automated the random assignment of medication numbers to medication packs containing each of the study drugs.

Patients, investigator staff, persons performing the assessments, and data analysts remained blinded to the identity of the treatment from the time of randomisation until database lock. A double-blind, double-dummy study design was used to conceal the identity of treatments by use of study drugs and respective matching placebos that were all identical in packaging, labelling, schedule of administration, and appearance.

### Patients

The study recruited male and female patients aged at least 40 years of age with symptomatic primary OA in the hip, knee, hand or spine (cervical or lumbar) for at least 3 months, and who required NSAID therapy that was expected to continue for 12 months (with possible single interruptions of no more than 4 weeks and total interruptions of no more than 3 months). Patients were also required to have a baseline pain assessment (5-point Likert scale) of mild, moderate or severe in the target joint.

Patients were excluded if they had: secondary OA with history and/or any evidence of rheumatoid arthritis (RA), uncontrolled gout, or inflammatory disease in the target joint; a history or evidence of infectious arthritis, pseudo-gout or acute forms of inflammatory gout in the target joint; hypersensitivity to analgesics, antipyretics or NSAIDs; peptic ulceration within the last 12 months or clinically significant GI bleeding within the last 5 years; hepatic (alanine aminotransferase [ALT] and/or aspartate aminotransferase [AST] >1.5 × upper limit of normal [ULN]; bilirubin >1.2 × ULN [unless consistent with Gilbert's disease in the opinion of the investigator]), renal, pancreatic or biliary disease, blood coagulation disorders, anaemia or platelet count of <100 × 10^9^/L; significant medical problems, such as uncontrolled hypertension, symptomatic heart failure; or any other clinically relevant condition or current medication that in the opinion of the investigator contra-indicates the use of any of the study or rescue medications. Pregnant or lactating women or premenopausal women not using an acceptable form of birth control were ineligible for inclusion, as were patients using: NSAIDs, except low-dose aspirin (75 mg-100 mg/day) if taking a stable dose for at least 4 weeks before randomisation and if not taken for secondary prevention of cardiovascular disease; systemic corticosteroids (except eye drops, topical or nasal application, or inhaled for asthma or chronic bronchitis taken at a stable dose for at least 2 weeks before randomisation); hyaluronic acid injection or intra-articular corticosteroid in the target joint in the last month; any drugs known to be contra-indicated with celecoxib (e.g. lithium, fluconazole, coumarins); or taking >2 g per day of rescue medication (acetaminophen/paracetamol) during the screening period. Before the protocol amendment, patients were ineligible if they had: an MI, stroke, coronary artery bypass grafting (CABG), invasive coronary revascularisation or new-onset angina within the 6 months before screening; or a history of coronary heart disease with electrocardiogram (ECG) evidence of silent MI, or congestive heart failure with symptoms at rest or with minimal activity or unstable angina. After the study had started, an amendment was made to the protocol following an announcement of possible increased CV risk with celecoxib. Consequently, patients with an elevated CV risk or a history of CV or cerebrovascular disease (i.e. angina pectoris of any severity, MI, CABG or percutaneous coronary intervention, transient ischaemic attack, clinically significant carotid artery stenosis or carotid endarterectomy, ischaemic stroke, or congestive heart failure NYHA class III-IV) were no longer eligible to participate in the study. Following the protocol amendment, patients taking low-dose aspirin for secondary prevention of cardiovascular disease were also discontinued, although those patients receiving low-dose aspirin for at least 4 weeks before screening for other reasons could continue.

### Study objectives and assessments

The study's primary objective was to demonstrate non-inferiority of lumiracoxib 100 mg o.d. or lumiracoxib 100 mg b.i.d. compared with celecoxib 200 mg o.d. with respect to the long-term retention rate in patients suffering from primary OA in hip, knee, hand or spine. The primary outcome variable was retention on treatment at 1 year; a patient was considered retained on treatment for 1 year if the study treatment was not discontinued before Week 50. As discontinuations can occur due to insufficient efficacy and/or safety or tolerability issues, retention of patients on treatment provides a measure of the overall effectiveness of the treatment. The secondary objectives of the study were to compare lumiracoxib versus celecoxib with respect to efficacy (OA pain intensity in the target joint; patient's global assessment of disease activity; physician's global assessment of disease activity), reasons for discontinuation, safety and tolerability, and patient-reported outcomes.

Efficacy was assessed by measuring the following parameters at baseline and at Weeks 4, 13, 20, 26, 39 and 52 using 5-point Likert scales: overall OA pain intensity in the target joint (1 = none, 2 = mild, 3 = moderate, 4 = severe, 5 = extreme); patient's global assessment of disease activity (1 = very good, 2 = good, 3 = fair, 4 = poor, 5 = very poor); physician's global assessment of disease activity (1 = very good, 2 = good, 3 = fair, 4 = poor, 5 = very poor). Patient-reported outcomes were analysed using the psychometric properties of the Short Arthritis assessment Scale (SAS). Patients completed the SAS, which comprises four 11-point scales (pain, global, difficulty with stairs and difficulty with shopping), at baseline and at Weeks 13, 26, 39 and 52.

Rescue medication use was recorded for the study duration. During the treatment phase of the study, a daily maximum of 2 g and a maximum cumulative dose of 2 g daily per two-thirds of the period between two consecutive visits was permitted. Patients exceeding this cumulative dose between visits were discontinued as having an unsatisfactory therapeutic effect.

Safety and tolerability were evaluated by recording adverse events (AEs) and serious AEs (SAEs) throughout the study. Physical examinations were performed at baseline and Weeks 13, 26, 39 and 52, and laboratory tests (haematology, blood chemistry, urinalysis) and vital signs were assessed at baseline and Weeks 4, 13, 20, 26, 39 and 52. Elevations in ALT and/or AST >3 × ULN were reported since this level was considered to be most relevant to health authorities and healthcare providers. Investigators were required to report all suspected predefined GI, CV/cerebrovascular and hepatic events for assessment and adjudication in a blinded manner by independent safety committees. The adjudicated CV/cerebrovascular events were assessed using a composite endpoint defined by the APTC, which includes CV death, MI and stroke (ischaemic or haemorrhagic) [24]. APTC events were reported up to 52 weeks after randomisation regardless of whether patients had discontinued prematurely (these patients had a follow-up call at 52 weeks). Deaths could be reported up until just before database lock and were included in the clinical database regardless of the patient's date of study completion or premature discontinuation. All SAEs occurring up to 30 days after the patient's last dose of study drug were reported to the Clinical Safety and Epidemiology department, according to standard Novartis SAE reporting procedures, and were not entered into the clinical database.

### Statistical analysis

Planned enrolment was for 750 patients in each of the lumiracoxib 100 mg o.d. and celecoxib 200 mg o.d. groups and 1,500 patients in the lumiracoxib 100 mg b.i.d. treatment arm. With these sample sizes, a one-sided test of proportions at the 1.25% level of significance had 98% power to show non-inferiority of lumiracoxib 100 mg b.i.d. to celecoxib 200 mg o.d., and 94% power to show non-inferiority of lumiracoxib 100 mg o.d. to celecoxib 200 mg o.d. Assumptions were made that the retention rates did not differ and were between 45% and 55% (a figure based upon previous experience with other lumiracoxib studies of 12 months' duration), and that the non-inferiority margin was -10%.

All efficacy evaluations were performed on the population of randomized patients who received at least one dose of study medication, the intention-to-treat (ITT) population. Non-inferiority of lumiracoxib for the primary outcome variable (1-year retention on treatment) was tested by a non-inferiority test comparing pairwise differences using a multiple testing procedure to adjust for multiplicity and a confidence interval (CI) approach with a predefined non-inferiority margin of -10%. The analysis was repeated for the per protocol population (all ITT patients without major protocol violations). The data were described by cumulative Kaplan-Meier estimates at Weeks 4, 13, 20, 26, 39 and 52. Retention on treatment was further explored by using a multiple logistic regression model with treatment as main effect and age as covariate. The reasons for discontinuation were also examined.

The secondary efficacy variables, overall OA pain intensity in the target joint, patient's global assessment of disease activity and physician's global assessment of disease activity, were summarised as the weighted average over the treatment period, representing the overall level of efficacy experienced, and evaluated using analysis of covariance (ANCOVA) with treatment as main effect and respective baseline value, age and centre as covariates. Results were presented as least squares means (LSM) with 95% CI. In addition, these variables were classified as 'improved', 'worsened' or 'unchanged' according to changes observed on the Likert scales from baseline to study endpoint; 'improvement' and 'worsening' were defined as a decrease and increase by at least one category, respectively. Improvement rates were compared between treatment groups by multiple logistic regression analysis (including treatment as main effect and age as covariate). Missing baseline assessments were replaced by the median of the baseline assessments and missing post-baseline data were imputed using the last observation carried forward (LOCF) technique. The four SAS scales were added to form the SAS total score. The analysis included questionnaires with no more than one missing question; missing values were replaced by the median value for all patients at the particular timepoint. The SAS total score was analysed using ANCOVA with treatment as main effect and baseline value, country and treatment as covariates. Rescue medication use was analysed using a multiple logistic regression model, with baseline pain intensity and treatment as main effects. All statistical tests for the secondary efficacy variables were performed at the 5% level of significance without adjusting for multiplicity.

Safety was evaluated in the safety population (all randomised patients who received at least one dose of study medication), which was identical to the ITT population. Safety data were analysed and presented using descriptive statistics.

After completion of the study and database lock, a quality assurance audit found that one of the participating centres failed to meet the required standards of clinical practice. Since the database had been locked including data from the 63 patients from this centre, a sensitivity analysis was performed to examine whether exclusion of this data would significantly change the outcomes from this study.

## Results

**Figure 1:**
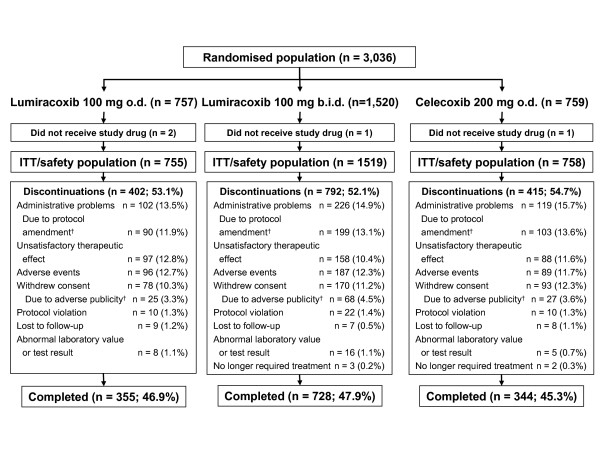
**Patient disposition**. ^†^Following an announcement of a possible increase in CV risk with celecoxib, some patients withdrew consent (4%) and some (12.9%) were discontinued after a protocol amendment excluded patients with an elevated CV risk or a history of CV or cerebrovascular disease; o.d. = once daily; b.i.d. = twice daily; ITT = intention-to-treat.

Between 07 September 2004 and 18 November 2004, a total of 3,036 patients were enrolled and randomised; four patients did not receive the study drug and were excluded from all analyses. Hence, a total of 755, 1,519 and 758 patients were included in the lumiracoxib 100 mg o.d., lumiracoxib 100 mg b.i.d. and celecoxib 200 mg o.d. treatment arms (ITT/safety population), respectively (Figure 1). The per protocol population comprised 648, 1310 and 675 patients randomized to the lumiracoxib 100 mg o.d., lumiracoxib 100 mg b.i.d. and celecoxib 200 mg o.d. treatment arms.

Baseline demographic and background characteristics were comparable across the treatment groups (Table 1). The majority of patients were female (71.0%), mean age was 62.5 ± 10.07 years and mean disease duration was 7.7 ± 7.73 years. The distribution of target OA joints assessed was 48.4% knee, 23.0% spine, 20.6% hand and 7.9% hip. In the majority of patients (93.3%), pain intensity in the target OA joint was moderate or severe.

**Table 1:** Baseline Patient Characteristics (ITT Population)

	**Lumiracoxib 100 mg o.d. (n = 755)**	**Lumiracoxib 100 mg b.i.d. (n = 1,519)**	**Celecoxib 200 mg o.d. (n = 758)**
Age (years), mean ± SD	62.9 ± 10.25	62.2 ± 10.02	62.7 ± 10.00
Females, n (%)	541 (71.7)	1081 (71.2)	531 (70.1)
BMI (kg/m^2^), mean ± SD	29.5 ± 6.41	29.7 ± 6.34	29.8 ± 6.33
Race, n (%)			
White/Caucasian	712 (94.3)	1450 (95.5)	731 (96.4)
Black/African American	22 (2.9)	45 (3.0)	13 (1.7)
Hispanic	10 (1.3)	8 (0.5)	11 (1.5)
Other^†^	11 (1.5)	16 (1.1)	3 (0.4)
Disease duration (years), mean ± SD	7.9 ± 7.73	7.6 ± 7.79	7.5 ± 7.62
Target joint, n (%)			
Hip	58 (7.7)	122 (8.0)	59 (7.8)
Knee	376 (49.8)	743 (48.9)	350 (46.2)
Hand	159 (21.1)	301 (19.8)	166 (21.9)
Spine	162 (21.5)	353 (23.2)	183 (24.1)
OA pain, n (%)			
Mild	48 (6.4)	102 (6.7)	49 (6.5)
Moderate	363 (48.1)	704 (46.3)	346 (45.6)
Severe	343 (45.4)	712 (46.9)	361 (47.6)
Extreme	1 (0.1)	1 (0.1)	2 (0.3)
Patient's global assessment of disease activity, n (%)			
Very good	2 (0.3)	8 (0.5)	4 (0.5)
Good	94 (12.5)	167 (11.0)	78 (10.3)
Fair	340 (45.0)	711 (46.8)	360 (47.5)
Poor	296 (39.2)	581 (38.2)	287 (37.9)
Very poor	23 (3.0)	51 (3.4)	29 (3.8)
Physician's global assessment of disease activity, n (%)			
Very good	1 (0.1)	0 (0.0)	2 (0.3)
Good	51 (6.8)	85 (5.6)	45 (5.9)
Fair	371 (49.1)	784 (51.6)	373 (49.2)
Poor	317 (42.0)	618 (40.7)	324 (42.7)
Very poor	14 (1.9)	31 (2.0)	12 (1.6)
Short Arthritis assessment Scale (SAS) total score, mean ± SD	22.4 ± 7.81	22.1 ± 7.61	22.3 ± 7.86

o.d. = once daily; b.i.d. = twice daily; BMI = body mass index; ITT = intention-to-treat; SD = standard deviation; ^†^Japanese, other Asian or Pacific Islander or other.

Patient disposition was also similar across the treatment groups. The percentage of patients completing the study was 46.9% for lumiracoxib 100 mg o.d., 47.9% for lumiracoxib 100 mg b.i.d. and 45.3% with celecoxib 200 mg o.d. (Figure 1). The main reasons for discontinuation were administrative problems (13.5–15.7%), AEs (11.7–12.7%), unsatisfactory therapeutic effect (10.4–12.8%) and withdrawal of consent (10.3–12.3%) (Figure 1). Following the protocol amendment excluding patients with an elevated CV risk or history of CV or cerebrovascular disease, 11.9–13.6% of patients were discontinued for administrative reasons and 3.3–4.5% withdrew consent.

### Retention on treatment

More than 45% of patients in each group remained on treatment for 1 year. Retention rates were 46.9% for lumiracoxib 100 mg o.d., 47.5% for lumiracoxib 100 mg b.i.d. and 45.3% with celecoxib 200 mg o.d. (Table 2). Treatment comparisons showed that retention rates were comparable for lumiracoxib 100 mg o.d. or lumiracoxib 100 mg b.i.d. compared with celecoxib 200 mg o.d. (Table 2). Results obtained from the per protocol population were consistent with these findings (estimated treatment differences between lumiracoxib 100 mg o.d. or lumiracoxib 100 mg b.i.d. and celecoxib 200 mg o.d. proportions were 0.02 [95% CI: -0.04, 0.08] and 0.02 [95% CI: -0.03, 0.07], respectively). Hence, retention on treatment with lumiracoxib at either dose was shown to be non-inferior to celecoxib 200 mg o.d. Descriptive comparisons of the two lumiracoxib groups demonstrated that lumiracoxib 100 mg o.d. was non-inferior to lumiracoxib 100 mg b.i.d.

**Table 2:** Treatment Comparisons of the Retention Rate at 1 Year (ITT Population)

**Treatment group**	**N**	**Retention rate, n (%)**	**Contrasts**	**Estimated difference (97.5% CI)**	**Outcome**
Lumiracoxib 100 mg	755	354 (46.9)	Lumiracoxib 100 mg o.d.	0.02 (-0.04, 0.07)	Non-inferior
o.d.			- celecoxib 200 mg o.d.		
Lumiracoxib 100 mg	1519	722 (47.5)	Lumiracoxib 100 mg b.i.d.	0.02 (-0.03, 0.07)	Non-inferior
b.i.d.			- celecoxib 200 mg o.d.		
Celecoxib 200 mg	758	343 (45.3)			
o.d.					

CI = confidence interval; o.d. = once daily; b.i.d. = twice daily; ITT = intention-to-treat.

**Figure 2:**
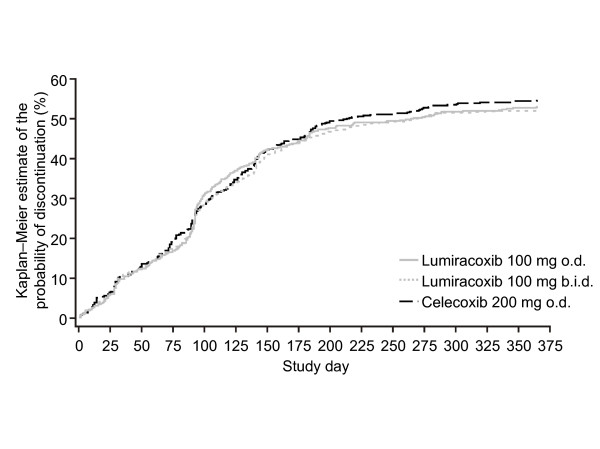
**Kaplan-Meier estimates of the probability (%) of premature discontinuation for any reason (ITT population)**. o.d. = once daily; b.i.d. = twice daily; ITT = intention-to-treat.

Kaplan-Meier curves for the probability of premature discontinuation from the study for any reason were similar for the three treatment groups (Figure 2). The median duration of exposure to study drug was lower for celecoxib (213.5 days) compared with lumiracoxib 100 mg o.d. and b.i.d. (266.0 and 268.0 days, respectively).

### Secondary efficacy variables

The LSM of the overall efficacy measures for the secondary efficacy variables were generally similar for all three treatments (Table 3). However, for physician's global assessment of disease activity, lumiracoxib 100 mg b.i.d. showed a small but statistically significant advantage in LSM over the other two treatments (2.45 vs 2.55 and 2.52 for lumiracoxib 100 mg o.d. and celecoxib 200 mg o.d.; p < 0.05). In addition, improvement rates at study end were comparable between the treatment groups for OA pain intensity, the patient's global assessment of disease activity and the physician's global assessment of disease activity (Table 3). No statistically significant differences between the treatments were observed.

**Table 3:** Improved OA Pain Intensity and Patient's and Physician's Global Assessments of Disease Activity (ITT Population)

	**Lumiracoxib 100 mg o.d. (n = 755)**	**Lumiracoxib 100 mg b.i.d. (n = 1,519)**	**Celecoxib 200 mg o.d. (n = 758)**
Patient's target joint pain intensity assessment			
Changes in scores from baseline to study endpoint, n (%)^†^			
Improvement	382 (50.6)	795 (52.3)	406 (53.6)
No change	269 (35.6)	566 (37.3)	265 (35.0)
Worsened	104 (13.8)	158 (10.4)	87 (11.5)
Overall measure of efficacy, least squares mean^‡^	2.78	2.72	2.77
Patient's global assessment of disease activity			
Changes in scores from baseline to study endpoint, n (%)^†^			
Improvement	368 (48.7)	768 (50.6)	373 (49.2)
No change	265 (35.1)	534 (35.2)	269 (35.5)
Worsened	122 (16.2)	216 (14.2)	116 (15.3)
Overall measure of efficacy, least squares mean^‡^	2.61	2.54	2.60
Physician's global assessment of disease activity			
Changes in scores from baseline to study endpoint, n (%)^†^			
Improvement	411 (54.5)	888 (58.5)	425 (56.2)
No change	244 (32.4)	463 (30.5)	232 (30.7)
Worsened	99 (13.1)	167 (11.0)	99 (13.1)
Overall measure of efficacy, least squares mean^‡^	2.55	2.45*	2.52

o.d. = once daily; b.i.d. = twice daily; ITT = intention-to-treat; ^†^patients with missing baseline values were not included; ^‡^Overall measure of efficacy defined as the weighted average of post-baseline scores using the last observation carried forward technique for missing values and time since previous visit as weight (lower scores represent better responses); *p < 0.05 vs lumiracoxib 100 mg o.d. and celecoxib 200 mg o.d.

Mean reductions from baseline in SAS scores occurred at all clinic visits for each of the treatment groups, demonstrating that patients had less pain, less difficulty going downstairs and shopping, and were doing better during treatment. There were no statistically significant differences between the groups at any timepoint (data not shown).

The percentage of patients who used rescue medication during the study was similar across treatment groups (lumiracoxib 100 mg o.d., 79.5%; lumiracoxib 100 mg b.i.d., 80.6%; celecoxib 200 mg o.d., 81.3%).

### Safety and tolerability

Both lumiracoxib doses were well tolerated, with similar incidences of AEs and SAEs reported in the three treatment groups (Table 4). Overall, AEs occurred in 72.6% of patients treated with lumiracoxib 100 mg o.d., 71.0% of patients receiving lumiracoxib 100 mg b.i.d. and 69.4% of patients receiving celecoxib 200 mg b.i.d. Infections and infestations were the most frequently reported AEs by primary system organ class (32.1–33.4%), followed by GI disorders (24.8–28.1%) and musculoskeletal and connective tissue disorders (21.8–23.3%). The 10 most frequent adverse events are listed in Table 5; headache (10.8–11.5%) and nasopharyngitis (9.3–10.6%) were the most common AEs in all treatment groups.

**Table 4:** Summary of AEs and SAEs (Safety Population)

	**Lumiracoxib 100 mg o.d. (n = 755)**	**Lumiracoxib 100 mg b.i.d. (n = 1,519)**	**Celecoxib 200 mg o.d. (n = 758)**
Patients with AEs, n (%)	548 (72.6)	1078 (71.0)	526 (69.4)
Patients with SAEs, n (%)	41 (5.4)	72 (4.7)	48 (6.3)
Fatal, n (%)^†^	2 (0.3)	7 (0.5)	1 (0.1)
Discontinuations due to AEs, n (%)	98 (13.0)	193 (12.7)	87 (11.5)

o.d. = once daily; b.i.d. = twice daily; AEs = adverse events; SAEs = serious adverse events; ^†^all deaths occurring before database lock are included.

**Table 5:** The 10 Most Frequent AEs (Safety Population)

	**Lumiracoxib 100 mg o.d. (n = 755)**	**Lumiracoxib 100 mg b.i.d. (n = 1,519)**	**Celecoxib 200 mg o.d. (n = 758)**
Headache	82 (10.9)	174 (11.5)	82 (10.8)
Nasopharyngitis	80 (10.6)	141 (9.3)	75 (9.9)
Back pain	46 (6.1)	87 (5.7)	52 (6.9)
Arthralgia	53 (7.0)	82 (5.4)	41 (5.4)
Abdominal pain upper	42 (5.6)	88 (5.8)	35 (4.6)
Upper respiratory tract infection	36 (4.8)	86 (5.7)	43 (5.7)
Dyspepsia	38 (5.0)	85 (5.6)	39 (5.1)
Urinary tract infection	38 (5.0)	75 (4.9)	24 (3.2)
Sinusitis	21 (2.8)	58 (3.8)	34 (4.5)
Influenza	30 (4.0)	53 (3.5)	28 (3.7)

o.d. = once daily; b.i.d. = twice daily.

SAEs occurred in 5.4% (n = 41) of patients treated with lumiracoxib 100 mg o.d., in 4.7% (n = 72) of patients receiving lumiracoxib 100 mg b.i.d. and 6.3% (n = 48) of patients receiving celecoxib 200 mg o.d. Overall, 10 deaths were reported before database lock (lumiracoxib 100 mg o.d., n = 2 [0.3%]; lumiracoxib 100 mg b.i.d., n = 7 [0.5%]; celecoxib 200 mg o.d., n = 1 [0.1%]). Of these, four patients died within 4 weeks of their last dose of study drug, two in the lumiracoxib 100 mg o.d. group (cerebrovascular accident [CVA] – not study drug-related in the investigator's opinion, haematemesis – study drug-related in the investigator's opinion) and two in the lumiracoxib 100 mg b.i.d. group (MI – not study drug-related in the investigator's opinion, CVA – study drug-related in the investigator's opinion). Of the other six deaths, which occurred more than 4 weeks after study drug discontinuation, five were in the lumiracoxib 100 mg b.i.d. group (CVA; CV failure secondary to pancreatic cancer; progression of anaplastic astrocytoma; angina/CV death; and sudden death/possible arrythmia) and one was in the celecoxib 200 mg o.d. group (probable CV death). Of these six deaths, only the angina/CV death in the lumiracoxib 100 mg b.i.d. group was considered to be study drug-related in the investigator's opinion.

The incidence of prespecified AEs (including GI events, GI ulcers, oedema, CV/cerebrovascular events and chest pain) was similar across treatment groups (lumiracoxib 100 mg o.d., 25.7%; lumiracoxib 100 mg b.i.d., 24.8%; celecoxib 200 mg o.d., 22.3%). GI events were the most common prespecified AEs (20–21% in the lumiracoxib groups, 18% with celecoxib).

Adjudicated GI, CV/cerebrovascular and liver events were uncommon with all three treatments. Adjudicated GI events (all suspected occurrences of perforations, ulcers and bleeding) occurred in 15 (2.0%) patients in the lumiracoxib 100 mg o.d. group, 17 (1.1%) in the lumiracoxib 100 mg b.i.d. group and 10 (1.3%) in the celecoxib 200 mg o.d. group. Symptomatic ulcers were observed in fewer patients treated with lumiracoxib 100 mg o.d. (n = 1 [0.1%]) or lumiracoxib 100 mg b.i.d. (n = 2 [0.1%]) compared with celecoxib 200 mg o.d. (n = 3 [0.4]). The incidence of definite or probable upper or lower GI tract ulcer complications was very low (lumiracoxib 100 mg o.d. 0.26%, lumiracoxib 100 mg b.i.d. 0.13% and celecoxib 200 mg o.d. 0.26%). None of the suspected upper GI tract complications were confirmed as definite by the independent safety committee. Probable upper GI tract ulcer complications were reported with lumiracoxib 100 mg b.i.d. (n = 1; haematochezia with signs of bleeding) and celecoxib 200 mg o.d. (n = 1; laboratory evidence of bleeding). The safety committee adjudicated two lower GI tract ulcer complications as definite (one case of haematochezia or melena resulting from bleeding in the large bowel reported as a suspected study drug-related SAE in the lumiracoxib 100 mg o.d. group, and one case of small bowel obstruction reported as an AE causing discontinuation in the celecoxib 200 mg o.d. group).

The number of patients with confirmed or probable CV/cerebrovascular events, defined using the APTC endpoint, was 3 (0.40%) with lumiracoxib 100 mg o.d., 6 (0.39%) with lumiracoxib 100 mg b.i.d. and 2 (0.26%) with celecoxib 200 mg o.d. The event analysis by time interval is shown in Table 6. The number of patients with confirmed or probable APTC events up to Week 52, regardless of study drug discontinuation, was 4 (0.53%) with lumiracoxib 100 mg o.d., 10 (0.66%) with lumiracoxib 100 mg b.i.d. and 5 (0.66%) with celecoxib 200 mg o.d.

**Table 6:** Time to events for elevations in hepatic transaminases and APTC events (Safety Population)

	**Lumiracoxib 100 mg o.d. (n = 755)**		**Lumiracoxib 100 mg b.i.d. (n = 1,519)**		**Celecoxib 200 mg o.d. (n = 758)**	
Time interval (days)	No. of subjects with events within the interval	Incidence rate within the interval (%)	No. of subjects with events within the interval	Incidence rate within the interval (%)	No. of subjects with events within the interval	Incidence rate within the interval (%)
*AST/ALT >3 × ULN*						
1 – 49	0	0.00	4	0.26	1	0.13
50 – 105	1	0.15	7	0.53	1	0.15
106 – 196	6	1.15	18	1.64	1	0.19
197 – 287	3	0.76	5	0.63	0	0.00
>287	1	0.28	1	0.14	0	0.00
*Definite/probable APTC events*						
1 – 49	0	0.00	1	0.07	0	0.00
50 – 105	0	0.00	1	0.07	1	0.13
106 – 196	1	0.13	1	0.07	0	0.00
197 – 287	1	0.13	3	0.20	1	0.13
>287	1	0.13	0	0.00	0	0.00

o.d. = once daily; b.i.d. = twice daily.

ALT/AST elevations >3 × ULN occurred at a higher frequency in patients treated with lumiracoxib 100 mg b.i.d. (twice the recommended dose) (n = 35 [2.3%]) than with lumiracoxib 100 mg o.d. (n = 11 [1.5%]) or celecoxib 200 mg o.d. (n = 3 [0.4%]). The type of injury was usually hepatocellular with some mixed and very few cases of pure cholestatic liver injury. The majority of patients affected were asymptomatic and none were clinically jaundiced. No 'Hy's' cases (ALT/AST >5 × ULN and bilirubin >3 mg/dL), which are more predictive for severe liver outcome, were observed with lumiracoxib 100 mg o.d., and one case was observed with lumiracoxib 100 mg b.i.d. after 143 days of treatment. An analysis by time intervals was performed and showed that, after short-term treatment (1–49 days), no cases of ALT/AST >3 × ULN were observed with lumiracoxib 100 mg o.d. (0.00%) compared with 4 (0.26%) with lumiracoxib 100 mg b.i.d. and 1 (0.13%) with celecoxib 200 mg o.d. (Table 6). The peak in the incidence of transaminase elevations with lumiracoxib was observed after 3 to 6 months of treatment (between 106 to 196 days) (Table 6) and then subsided at later timepoints. After >287 days of treatment, the incidence rates of ALT/AST <3 × ULN decreased to 0.28% with lumiracoxib 100 mg o.d. and 0.14% with lumiracoxib 100 mg b.i.d.). Patients with an AST and/or ALT value between greater than 3 × ULN and less than 5 × ULN could continue on treatment but liver function tests were to be repeated within 2 weeks and patients with a value greater than or equal to the previous value at repeat testing were immediately discontinued. An AST and/or ALT value of between greater than 3 × ULN and less than 5 × ULN was reported in 1.1% of the lumiracoxib 100 mg o.d. group, 0.8% of the lumiracoxib 100 mg b.i.d. group and 0.4% of the celecoxib 200 mg o.d. group; the median times to normalization, i.e. when both AST and ALT had returned to less than 2 × ULN, were 16, 20 and 14 days, respectively. Patients with elevated liver enzymes were discontinued from treatment if AST or ALT values were greater than 5 × ULN, which occurred in 0.4% of the lumiracoxib 100 mg o.d. group, 1.8% of the lumiracoxib 100 mg b.i.d. group and no patients in the celecoxib 200 mg o.d. group. The time to normalization for ALT/AST values > 5 × ULN ranged between 4 and 132 days. For those patients who discontinued due to abnormal liver function tests, follow-up values were available for all but one patient (who died due to a cerebrovascular accident) and transaminase levels were consistently shown to return to normal or close to normal. Of note, a small number of patients in each treatment group had abnormal liver function tests (ALT/AST >1.5 ULN or bilirubin >1.2 × ULN) at screening and should have been excluded: 0.9% of patients in the lumiracoxib 100 mg o.d. group, 0.8% of the lumiracoxib 100 mg b.i.d. group and 0.3% in the celecoxib 200 mg o.d. group.

The incidences of notable abnormalities of haematology, biochemistry, urinalysis and vital signs were low and generally similar across treatment groups. New-onset changes in renal function (>35.36 μmol/L [0.4 mg/dL] increase in creatinine level from baseline was chosen as a marker for renal AEs by the Data Safety Management Board [DSMB]) occurred in 16 patients (2.1%) treated with lumiracoxib 100 mg o.d., 26 patients (1.7%) receiving lumiracoxib 100 mg b.i.d. and five patients (0.7%) treated with celecoxib 200 mg o.d. Notable increases in blood pressure, pulse or weight tended to occur less frequently with lumiracoxib 100 mg o.d. than in the other two groups. Mean changes from baseline in systolic blood pressure were -2.2 mmHg with lumiracoxib 100 mg o.d., 0.1 mmHg with lumiracoxib 100 mg b.i.d. and -0.3 mmHg with celecoxib 200 mg o.d. The corresponding changes from baseline for diastolic blood pressure were -1.1%, -0.5% and -0.1%. No patients in the lumiracoxib 100 mg o.d. group experienced a notable increase in pulse rate (increase from baseline of ≥ 15% and value of ≥ 110 bpm) compared with 0.2% and 0.3% in the lumiracoxib 100 mg b.i.d. and celecoxib 200 mg o.d. groups, respectively. Weight increases of over 5% from baseline were reported by 10.2% of the lumiracoxib 100 mg o.d. group, 13.8% of the lumiracoxib 100 mg b.i.d. group and 11.2% of the celecoxib 200 mg o.d. group.

### Sensitivity analysis

In the sensitivity analysis excluding data from the centre with major violations of GCP, there were no clinically relevant differences from the total ITT population for any efficacy variable. One-year retention rates for the ITT population excluding the 63 patients from this centre were comparable with those for the total ITT population, and there were no differences between these populations in Kaplan-Meier estimates for the probability of premature discontinuation from the study for any reason.

A total of 10 of the 63 patients from the excluded centre reported AEs. Overall, proportions of patients with any AE or with specific AEs were similar to the total safety population. However, two of the patients from this centre were reported to have notable increases in liver function tests. Excluding the centre that was audited, the incidence of ALT/AST elevations (>3 × ULN) probably or possibly related to study drug was 1.4% with lumiracoxib 100 mg o.d., 2.3% with lumiracoxib 100 mg b.i.d. (twice the recommended dose), and 0.4% with celecoxib 200 mg o.d.

## Discussion

This large, randomised, double-blind study has demonstrated that lumiracoxib 100 mg o.d., the recommended dose for OA, was non-inferior to celecoxib 200 mg o.d. for retention on treatment at 1 year in patients with OA of the knee, hip, hand and spine. As this outcome variable is dependent on the number of treatment discontinuations, which mainly occur for insufficient efficacy and tolerability issues, it indicates that the overall efficacy and safety of lumiracoxib 100 mg o.d. is comparable with that of celecoxib 200 mg o.d. Similar findings were observed with lumiracoxib at the higher dose of 100 mg b.i.d. Previous studies have reported that discontinuation rates are lower with selective COX-2 inhibitors compared with traditional NSAIDs, such as ibuprofen and naproxen [23,25]. These observations could suggest that COX-2 inhibitors have a more favourable balance of both efficacy and tolerability compared with NSAIDs.

Both doses of lumiracoxib were associated with improvements similar to celecoxib in all secondary efficacy parameters. At Week 52, approximately 50% of patients in each treatment group assessed their OA target joint pain and disease activity to be reduced, with over half of physicians also reporting lower disease activity. SAS scores were improved in all three treatment groups and there were no statistically significant between-group differences in the use of rescue medication. Although the use of rescue medication was high across all three treatment groups, this would not be unexpected in a 1-year study.

Lumiracoxib 100 mg o.d. was also shown to be as well tolerated as celecoxib 200 mg o.d. in this large group of patients with OA over 1 year. The overall incidence and type of AEs for lumiracoxib at both doses were comparable with those observed with celecoxib 200 mg o.d., and as expected given the duration of the study and the population studied.

The incidence of adjudicated CV/cerebrovascular events was very low across the treatment arms, supporting previous findings that lumiracoxib has a low risk of CV events that is comparable with NSAIDs. In TARGET, the incidence of non-fatal and silent MI, stroke, or CV death with lumiracoxib 400 mg o.d. was comparable with that observed with traditional NSAIDs, ibuprofen and naproxen [26]. Moreover, a meta-analysis of lumiracoxib studies with 34,668 patients has also reported that the risk of CV events with lumiracoxib was not statistically significantly different from placebo, naproxen or non-naproxen NSAIDs [27]. These findings are in keeping with a recent meta-analysis, which has also indicated that the risk of CV events with selective COX-2 inhibitors is similar to that observed with most NSAIDs [28]. Another systematic review and meta-analysis of observational studies has noted some differences in CV risk with individual NSAIDs and selective COX-2 inhibitors: there was no elevation in CV risk with celecoxib, naproxen or ibuprofen, but rofecoxib and diclofenac were associated with a significantly increased risk of serious CV events [29]. These findings support lumiracoxib as a selective COX-2 inhibitor with a CV risk profile comparable with most other NSAIDs. Other studies have indicated that lumiracoxib has a blood pressure profile similar to placebo [30] and superior to traditional NSAIDs [26,31], which support its CV safety profile.

Lumiracoxib is indicated at a dose of 100 mg o.d. for chronic use in OA, and at doses of 200 mg or 400 mg o.d. for short-term use in acute pain indications. While liver toxicity is a known rare but serious side effect of all COX-2 inhibitors and traditional NSAIDs [32], there have been some specific concerns from health authorities regarding the hepatic profile of lumiracoxib. Australia withdrew lumiracoxib in August 2007 following reports of severe liver events occurring predominantly at doses higher than lumiracoxib 100 mg o.d. taken chronically. The US FDA issued a non-approvable letter in September 2007, citing concerns over the hepatic profile of lumiracoxib. This was followed by withdrawals in Canada, Europe and a few other countries. Assessment of the benefit-risk profile of the drug is ongoing by a number of health authorities. In this analysis, elevations in liver enzymes of more than 3 × ULN, submitted for adjudication and considered to be related to the study drug, occurred in 1.5% of patients treated with lumiracoxib 100 mg o.d. This incidence is greater than previously observed for ALT/AST elevations >3 × ULN with lumiracoxib 100 mg o.d. in long-term clinical trials (0.91%) (Novartis: data on file, Studies 2360 [core plus extension] and 2361 [core plus extension] pooled). However, the incidence rate for lumiracoxib 100 mg o.d. is similar to that reported in the prescribing information for many NSAIDs (1% or less), such as naproxen and ibuprofen [33,34], and less than that observed with diclofenac, which has been associated with ALT and AST abnormalities in 3.2% and 1.8% of patients, respectively [35]. It is also important to note that it is very rare for the elevations in liver enzymes with NSAIDs to translate into SAEs [36]. Moreover, as shown in this study, elevations in liver function tests are generally reversible when treatment is discontinued. Given the increased incidence of ALT/AST elevations and the lack of additional efficacy benefit with lumiracoxib 100 mg b.i.d., it would not be advisable to exceed the recommended dose of lumiracoxib (100 mg o.d.) for the long-term treatment of OA.

Given that OA pain may require treatment over extended periods of time, these findings demonstrating that lumiracoxib is efficacious and well tolerated over 1 year are important. The long-term efficacy, safety and tolerability profile of lumiracoxib 100 mg o.d. compared with celecoxib 200 mg o.d. has also been evaluated previously in a 39-week, double-blind extension to a 13-week, multicentre, randomised, double-blind, placebo-controlled trial of patients with knee OA [16,37]. Comparable improvements were seen for both treatments at all timepoints in all three efficacy variables: OA pain intensity, patient's global assessment of disease activity and Total score of the Western Ontario and McMaster Universities (WOMAC™) Osteoarthritis Index LK 3.1 questionnaire [16,37]. Furthermore, the incidence and type of AEs reported were similar between celecoxib 200 mg o.d. and lumiracoxib 100 mg o.d. [16,37] and elevations in liver enzymes were in line with previously reported incidence rates with NSAIDs.

One limitation of this study was related to an announcement after the study had started that there may be a possible increase in CV risk with celecoxib. To ensure patient safety, a protocol amendment to patient eligibility was implemented and 17% of randomised patients, who had an elevated CV risk, had to be discontinued. Although this accounted for a significant proportion of treatment discontinuations, similar to that attributed to insufficient efficacy or AEs, supportive analyses showed that this did not affect the relative distribution for retention on treatment across the treatment arms (data not shown). A second limitation of this study was the inclusion of patients from a centre that did not meet the required standards of clinical practice. However, a sensitivity analysis demonstrated that exclusion of data from this centre resulted in the efficacy and safety data similar to that observed for the total ITT population.

## Conclusion

In conclusion, these data show that lumiracoxib 100 mg o.d. was as effective and well tolerated as celecoxib 200 mg o.d. during long-term treatment of up to 1 year in patients with OA. Hence, lumiracoxib should be considered as a useful treatment option for the relief of OA pain.

## List of abbreviations used

AEs, Adverse events; ALT Alanine aminotransferase; ANCOVA, Analysis of covariance; APTC, Antiplatelet Trialists' Collaboration; AST, Aspartate aminotransferase; b.i.d., Twice daily; BMI, Body mass index; CI, Confidence interval; COX-1, Cyclooxygenase-1; COX-2, Cyclooxygenase-2; CV, Cardiovascular; CVA, Cerebrovascular accident; DSMB, Data Safety Management Board; ECG, Electrocardiogram; GCP, Good Clinical Practice; GI, Gastrointestinal; ITT, Intention-to-treat; LOCF, Last observation carried forward; LSM, Least squares means; MEDAL, Multinational Etoricoxib and Diclofenac Arthritis Long-term; MI, Myocardial infarction; NSAIDs, Nonsteroidal anti-inflammatory drugs; OA, Osteoarthritis; o.d., Once daily; RA, Rheumatoid arthritis; SAEs, Serious adverse events; SAS, Short Arthritis assessment Scale; SD, Standard deviation; TARGET, Therapeutic Arthritis and Gastrointestinal Event Trial; ULN, Upper limit of normal; WOMAC™, Western Ontario and McMaster Universities

## Competing interests

Neha P Patel is an employee of, and owns shares in, Novartis Pharmaceuticals Corporation, East Hanover, NJ, USA. Marianne Notter and Peter Sallstig are employees of, and own shares in, Novartis Pharma AG, Basel, Switzerland.

Jean-Yves Reginster has received consulting fees from Servier, Novartis, Negma, Lilly, Wyeth, Amgen, GlaxoSmithKline, Roche, Merckle, Nycomed, NPS, Theramex, lecturing fees from Merck Sharp and Dohme, Lilly, Rottapharm, IBSA, Genevrier, Novartis, Servier, Roche, GlaxoSmithKline, Teijin, Teva, Ebewee Pharma, Zodiac, Analis, Theramex, Nycomed, Novo-Nordisk, and research grant support from Bristol Myers Squibb, Merck Sharp & Dohme, Rottapharm, Teva, Lilly, Novartis, Roche, GlaxoSmithKline, Amgen, Servier.

Roy Fleischmann has received research grant support from Novartis, Pfizer, Amgen, Wyeth, Abbott, Centocor, Genentech, Roche, Lilly, USB and TAP. He has been a consultant for Novartis, Pfizer, Amgen, Wyeth, Abbott, Centocor, Genentech, Roche, USB and Lilly, He is a member of the Speakers Bureau for Amgen, Wyeth, Abbott, Genentech and Roche.

Hyman Tannenbaum has received lecture and/or consulting fees from Amgen, Bristol Myers Squibb, Merck-Frosst Canada, Novartis Pharmaceuticals, Pfizer, and Wyeth.

## Authors' contributions

J-YR, RF and HT were all investigators. PS was the Program Section Leader Phase III (having overall responsibility for the conduct of the trial and overseeing the correctness of the outputs), NPP was the study Clinical Trial Leader and was involved in the conduct of the trial, and both were involved in the design of the trial. MN was the Trial Statistican, and was involved in study design and analysis of the data. All authors contributed to the development of the manuscript.

## Appendix: List of Investigators

**Belgium: **Dr F. d'Argent, Private Practice, Comines; Dr R. Leliaert, Private Practice, Tielt; Dr G. Mehuys, Private Practice, Tielt; Prof Dr J-Y Reginster, CHU Policlinique L.Brull, Liège; Dr H. Van Aerde, Private Practice, Genk; Dr P. Van Belle, Private Practice, Kraainem; Dr D. Vanroyen, Private Practice, Hasselt; Dr M. Veevaete, Private Practice, Bruxelles; Dr J. Vernijns, Private Practice, Genk; Dr G. Watté, Private Practice, Tielt; Dr M. Wouters, CHIREC – Clinique du Parc Léopold, Bruxelles.

**Canada: **Dr M. Awde, Murray Awde Medicine Professional Corporation, London, ON; Dr A. Bailey, BioQuest Research, Spruce Grove, AB; Dr A. Beaulieu, Clinique Médicale St.-Louis, Ste-Foy, QC; Dr M. Bell, Sunnybrook & Women's College Health Sciences Centre, Toronto, ON; Dr W. Bensen, WynnTech Inc., Hamilton, ON; Dr W. Booth, Antigonish Clinical Trials, Antigonish, NS; Dr L. Breger, Kells Medical Research Group, Pointe-Claire, QC; Dr B. Carlson, North Road Medical Centre, Coquitlam, BC; Dr A. Chow, Credit Valley Prof Bldg, Mississauga, ON; Dr A. Cividino, Mac Research Inc., Hamilton, ON; Dr H. Conter, MSHJ Research Associates Inc., Halifax, NS; Dr D. Craig, Dr Donald Craig, St John, NB; Dr E. Dessouki, ADA Medical Ltd, Oshawa, ON; Dr L. Ferguson, Colchester Research Group, Truro, NS; Dr G. Girard, Novabyss Inc., Sherbrooke, QC; Dr B. Haraoui, Institut de Rhumatologie de Montreal, Montreal, QC; Dr R. Hart, White Hills Medical Clinic, St John's, NF; Dr E. Howlett, Lenore Centre Medical Centre, Saskatoon, SK; Dr C. Hudon, Unité de médecine de famille, Chicoutimi, QC; Dr N. Hudson, PCT Networks Inc., Kelowna, BC; Dr S. Jaffer, Dr Shahin Jaffer Inc., Delta, BC; Dr J. Kooy, PCT Networks Inc., Penticton, BC; Dr M. Lafreniere, Clinique Medicale Pierre Bertrand, Vanier, QC; Dr S.Y. Lam, HAWSE Clinic, Calgary, AB; Dr B. Lasko, Manna Research, Toronto, ON; Dr J. Li, G.A. Research Associates Ltd, Moncton, NB; Dr F. Morin, Centre de Recherche Musculo-Squelettique, Trois-Rivieres, QC; Dr W. Olsheski, Albany Medical Clinic, Toronto, OH; Dr M. O'Mahony, London Road Diagnostic Clinic and Medical Centre, Sarnia, ON; Dr M. Omichinsky, Portage Clinical Studies, Portage La Prairie, MB; Dr J.-P. Ouellet, Q&T Research, Inc., Sherbrooke, QC; Dr B. Pynn, Clinical Research Consultant Group, Beaconsfield, QC; Dr B. Ramjattan, First Line Medical Services Ltd, St John's, NF; Dr J. Rodrigues, Dr Jude Rodrigues, Windsor, ON; Dr K. Saunders, McPhillips Medical Clinic, Winnipeg, NB; Dr K. Skeith, Allin Clinic, Edmonton, AB; Dr R. Somani, Dr Rizman Somani, Langley, BC; Dr E. St-Amour, Q&T Research, Inc., Gatineau, QC; Dr H. Tannenbaum, Rheumatic Disease Centre of Montreal, Montreal, QC; Dr J. Tannenbaum, Meadowlands Family Health Center, Ottawa, ON; Dr M. Tolszcuk, Q&T Research, Inc., Sherbrooke, QC; Dr W. Yang, Allergy and Asthma Research Centre, Ottawa, ON; Dr B. Zidel, Malton Medical Centre, Mississauga, ON; Dr M. Zummer, Polyclinique Maisonneuve-Rosemont, Montreal, QC.

**France: **Dr P. Beignot Devalmont, Cabinet du Dr Beignot Devalmont, Rouen; Dr L. Boucher, Cabinet du Dr Boucher, Murs Erigné; Dr A. Campagne, Cabinet du Dr. Alain Campagne, Tours; Dr B. Chagnoux, Cabinet du Dr Chagnoux, Bourges; Dr J.-B. Churet, Cabinet du Dr Churet, Le Pradet, France; Dr A. El Sawy, Cabinet du Dr El Sawy, St. Martin d'Heres; Dr F. Liotard, Private Practice, Laragne; Dr C. Magnani, Cabinet du Dr Magnani, L'Aigle; Dr S. Musso, Cabinet du Dr Musso, Eaunes; Dr D. Pineau Valencienne, Cabinet du Dr Pineau Valencienne, Nantes; Dr J. Sicard, Cabinet Médical; St. Romain/Cher; Dr F. Spilthooren, Cabinet du Dr. François Spilthooren, Evreux.

**Germany: **Dr C. Fleige, Klinische Forschung Berlin Mitte, Berlin; Dr A. Herzner, Klinische Forschung Hamburg, Hamburg; Dr R. Lehmann, Klinische Forschung Berlin-Buch GmbH, Berlin; R. Nischick (Dipl Med), Zentrum Therapiestudien/Dr Nischick, Leipzig; Dr H. Schneider, Praxis Dr. Schneider, Bad Nauheim; Dr M. Schreinert, Klinische Forschung Berlin Mitte, Berlin; Dr V. von Behren, Praxis Dr von Behren, Wiesbaden.

**Italy: **Prof S. Adami, Centro Ospedaliero Clinicizzato di Medicina Riab. e Prev., Valeggio Sul Mincio; Professor E. Ambrosioni, Az. Osp. di Bologna Policl. S. Orsola – Malpighi, Bologna; Dr S. Bernini, Policlinico – Università degli Studi, Modena; Dr G. Bianchi, Az. San. Genovese 3 P.O. Genova Ponente – S.O. La Colletta, Arenzano; Prof S. Bombardieri, Az. Osp. Ospedali Riuniti S. Chiara – Università degli Studi, Pisa; Prof C. Borghi, Az. Osp. di Bologna Policl. S. Orsola – Malpighi, Bologna; Dr M. Broggini, Ospedale di Circolo Fondazione Macchi, Varese; Prof M. Carrabba, Ospedale Luigi Sacco – Azienda Ospedaliera, Milano; Prof M. Cipriani, Presidio Ospedaliero della Misericordia, Grosseto; Prof C. Ferri, Az. Ospedal. Univ. di Modena Policlinico Univ. degli Studi, Modena; Prof W. Grassi, Presidio Ospedaliero Ospedale Murri, Jesi; Dr P. Manganelli, Ospedale Maggiore – Azienda Ospedaliera di Parma, Parma; Prof U. Martorana, Policlinico Universitario, Università degli Studi di Palermo, Palermo; Prof M. Matucci Cerinic, Azienda Ospedaliera Careggi – Università degli Studi, Firenze; Prof S. Minisola, Azienda Policlinico Umberto I° – Università La Sapienza, Roma; Prof C. Montecucco, Policlinico S. Matteo – IRCCS Università degli Studi di Pavia, Pavia; Dr M. Muratore, Presidio Ospedaliero Galateo, San Cesario di Lecce; Prof I. Olivieri, Azienda Ospedaliera S. Carlo, Potenza; Dr L. Punzi, Azienda Ospedaliera di Padova – Università degli Studi, Padova; Prof G. Rovetta, Istituto E. Bruzzone, Genova; Dr S. Scarpato, Ospedale Scarlato ASL Salerno 1, Scafati; Prof B. Seriolo, Azienda Osp. Ospedale S. Martino – Università degli Studi, Genova; Prof G. Valesini, Azienda Policlinico Umberto I° – Università La Sapienza, Roma.

**Switzerland: **Dr H. Fahrer, Praxis im Lindenhofspital, Bern; Dr T. Lehmann, Praxis Dr Lehmann, Bern; Dr M. Pellaton, Praxis Dr Pellaton, Neuchâtel; Dr R. Theiler, Stadtspital Triemli, Zürich; Prof A. Tyndall, Felix Platter Spital, Basel; Dr D. Uebelhart, Universitätsspital Zürich, Zürich; Prof P.M. Villiger, Inselspital Bern, Bern.

**USA: **Dr E. Arnold, Illinois Bone & Joint Institute, Morton Grove, IL; Dr A. Aven, Clinical Research Associates, Ltd, Arlington Heights, IL; Dr S. Baumgartner, The Physicians Clinic of Spokane, Spokane, WA; Dr D. Benson, DMI Healthcare Group, Inc., Largo, FL; Dr E. Boling, Boling Clinic Trials, Upland, CA; Dr S. Bookbinder, Ocala Rheumatology Research Center, Ocala, FL; Dr D. Borenstein, Arthritis and Rheumatism Associates, Washington, DC; Dr J. Box, The Arthritis Clinic at Carolina Bone & Joint Center, Charlotte, NC; Dr D. Brandon, California Research Foundation, San Diego, CA; Dr P. Buchanan, River Road Medical Group, Eugene, OR; Dr F. Burch, Radiant Research, San Antonio, TX; Dr. B. Caciolo, St. Louis Center for Clinical Research, St Louis, MO; Dr J. Cato, Dial Research Associates, Nashville, TN; Dr V. Chindalore, Pinnacle Research Group, LLC, Anniston, AL; Dr J. Christensen, Nevada Access to Research & Education Society, Las Vegas, NV; Dr D. Colan, Internal Medicine Associates, Grand Island, NE; Dr J. Condemi, AAIR Research Center, Rochester, NY; Dr B. Corser, Community Research, Cinncinnati, OH; Dr R. Craven, East Coast Clinical Research, LLC, Virginia Beach, VA; Dr A. Dahdul, FutureCare Studies, Springfield, MA; Dr D. Dayon, Adviso Medical Research, LLC, Chicago, IL; Dr M. Dewan, Meera Dewan, P.C., Omaha, NE; Dr A. Dikranian, San Diego Arthritis & Osteoporosis Medical Clinic, San Diego, CA; Dr S. Elliott, MediSphere Medical Research Center, Evansville, IN; Dr R. Ettlinger, Cedar Medical Center, Tacoma, WA; Dr S. Fallahi, Montgomery Rheumatology Associates, Montgomery, AL; Dr F. Farmer, Radiant Research Daytona Beach, Daytona Beach, FL; Dr C. Fisher, Health Research of Hampton Roads, Newport News, VA; Dr D. Fiske, Clinical Research Center of South Florida, Stuart, FL; Dr R. Fleischmann, Radiant Research, Dallas, Texas; Dr S. Folkerth, Clinical Research Center of Nevada/Summit Medical Group, Las Vegas, NV; Dr J. Gresh, Renstar Medical Research, Ocala, FL; Dr M. Grisanti, Buffalo Rheumatology, Orchard Park, NY; Dr W. Harper, Wake Research Associates, Raleigh, NC; Dr E. Harris, E. Robert Harris Medical Corporation, Whittier, CA; Dr D. Haselwood, Med Investigations, Fair Oaks, CA; Dr M. Heick, The Physicians Clinic of Spokane, Spokane, WA; Dr J. Herrod, Cochise Clinical Research, Sierra Vista, AZ; Dr J. Huff, Arthritis Center South Texas, San Antonio, TX; Dr D. Jones III, Alabama Research Center, Birmingham, AL; Dr J. Kaine, Sarasota Arthritis Center, Sarasota, FL; Dr R. Karr, Physicians Pharmaceutical Study Services, Everett, WA; Dr B. Kerzner, Health Trends Research, Baltimore, MD; Dr H. Knapp, Deaconess Billings Clinic Research Division, Billings, NT; Dr M. Kohen, Coastal Medical Research Center, Port Orange, FL; Dr W. Larson, Radiant Research, Lakewood, WA; Dr J. LaSalle, Medical Arts Research Collaborative, Excelsior Springs, MO; Dr D. Lee, Irvine Center for Clinical Research, Irvine, CA; Dr J.D. Lehmann, Ridgeview Research Center, Chaska, MN; Dr R. Lipetz, Encompass Clinical Research, Spring Valley, CA; Dr T. Littlejohn, Piedmont Medical Research Associates, Inc., Winston-Salem, NC; Dr J. Loveless, Intermountain Orthopedics, Boise, ID; Dr B. Lubin, Hampton Roads Center for Clinical Research, Norfolk, VA; Dr N. Lunde, Twin Cities Clinical Research, Brooklyn Park, MN; Dr F. Maggiacomo, New England Center for Clinical Research, Cranston, RI; Dr H. Mcilwain, Tampa Medical Group, P.A., Tampa, FL; Dr J. McKay, Oklahoma Ctr for Arthritis Therapy & Research, Tulsa, OK; Dr E. McPherson, Clinical Research of Winston-Salem, Winston-Salem, NC; Dr K. Miller, Arthritis Associates of CT/NY, LLC, Danbury, CT; Dr S.D. Miller, Northeast Medical Research Associates, Inc., North Dartmouth, MA; Dr V.J. Mirkil, Clinical Research Consortium, Las Vegas, NV; Dr A. Mollen, Southwest Health, Ltd, Phoenix, AZ; Dr M. Morgan, Complete Family Care, Northglenn, CO; Dr D. Moss, Carolinas Research Associates, Charlotte, NC; Dr. N. Neal, Valerius Medical Group and Research Center of Long Branch, Long Beach, CA; Dr T. Nolen, TOMAC, Inc., Columbiana, AL; Dr D. Noritake, Pasadena, CA; Dr D. Petrone, Research Associates of NorthTexas, Dallas, TX; Dr E. Portnoy, Westlake Medical Research, Westlake Village, CA; Dr A. Puopolo, Milford Emergency Associates, Inc., Milford, MA; Dr B. Rankin, University Clinical Research – DeLand, DeLand, FL; Dr K. Rock, Greystone Medical Research, LLC, Birmingham, AL; Dr S. Rosenblatt, Irvine Center for Clinical Research, Irvine, CA; Dr J. Rubino, Triangle Medical Research Associates, Raleigh, NC; Dr B. Sakran, Southern Illinois Clinical Research Centre, O'Fallon, IL; Dr M. Sayers, Arthritis Associates of Colorado Springs, Colorado Springs, CO; Dr J. Schechtman, Sun Valley Arthritis Center, Ltd, Glendale, AZ; Dr D. Schoenwalder, Mercy Med Group – Woodlake Research, Chesterfield, MO; Dr. C. Scoville, Insitute of Arthritis Research, Idaho Falls, ID; Dr A. Sebba, Arthritis Associates, Palm Harbor, FL; Dr S. Sharp, Clinical Research Associates, Nashville, TN; Dr Y. Sherrer, CRIA Research, Ft Lauderdale, FL; Dr T. Shlotzhauer, Rochester Clinical Research, Rochester, NY; Dr B. Short, Pinnacle Medical Research, Overland Park, KS; Dr T. Smith, Mercy Health Research, St Louis, MO; Dr C. Strout, Coastal Carolina Research Center, Mount Pleasant, SC; Dr R. Surowitz, Health Awareness, Jupiter, FL; Dr J. Tesser, Radiant Research – Phoenix North, Phoenix, AZ; Dr H. Thomas, CTT Consultants, Inc., Prairie Village, KS; Dr M. Tonkon, Apex Research Institute, Santa Ana, CA; Dr R. Trapp, The Arthritis Center, Springfield, IL; Dr R. Valente, Arthritis Center of Nebraska, Lincoln, NE; Dr R. Wade, Progressive Clinical Research, Centerville, UT; Dr W. Warnes, Meridian Clinical Research, Omaha, NE; Dr S. Weisman, Boulder Medical Center, Boulder, CO; Dr R. Weltman, Physician's Research Center, Hartford, CT; Dr C. Wiesenhutter, Coeur d'Alene Arthritis Clinic, Coeur d'Alene, ID; Dr M. Wiggins, Clinical Research Coordinating Svc., Loveland, CO; Dr R. Williams, Georgia Clinical Professionals Group, Athens, GA; Dr S. Williams, PharmaTex Research LLC, Amarillo, TX; Dr L. Willis, Lynn Health Science Institute, Oklahoma City, OK; Dr M. Wukelic, Rockwood Clinic, Spokane, WA; Dr D. Zmolek, Central New York Clinical Research, Manlius, NY.

## Pre-publication history

The pre-publication history for this paper can be accessed here:


